# Understanding Parent–school Communication for Students with Emotional and Behavioral Disorders

**DOI:** 10.2174/1874922401709010122

**Published:** 2017-10-10

**Authors:** Rohanna Buchanan, Miriam Clark

**Affiliations:** Oregon Social Learning Center, Eugene, 97401, Oregon, USA

**Keywords:** EBD, Parent involvement, Special education, Transitions, Barriers, Communication

## Abstract

**Background:**

Parents of children with Emotional and Behavioral Disorders (EBD) often face barriers to effective communication with schools. They often feel blamed or stigmatized for their children’s behavior and, while advocating, can feel adversarial with the school.

**Objective:**

The current study aims to describe current communication for parents and teachers of students with EBD, identify parent–school communication barriers, and identify characteristics of effective parent–school communication.

**Method:**

We conducted 15 semi-structured, qualitative interviews with parents and teachers of students with EBD. Interview questions focused on descriptions of the tone of their current communication, perceived barriers to communication, and ideas for effective approaches to communication. All interviews were audio-recorded, transcribed verbatim, and coded.

**Results:**

Results show that parents’ feelings about their current parent–school communication varied by the type of school students were attending: day-treatment, neighborhood, military charter, or private treatment school. Both parents and teachers brought up complex issues with parent–school communication related to school culture and the impact of parents’ prior negative experiences communicating with schools. Parents and teachers identified barriers to communication and suggestions for effective communication related to technology, institutional issues, time, parents’ and teachers’ lack of interest in communicating, and school proximity to home.

**Conclusion:**

Parents and teachers identified parent–school communication as a critical factor to promote children’s school success. Three broad recommendations emerged from the interviews targeting malleable factors to increase effective parent–school communication. We also discuss limitations and implications for practice.

## 1. INTRODUCTION

Ample research shows the benefits of a strong parent–school relationship. Parent–school involvement is shown to increase child self-efficacy for learning [1 – 3], raise child perceptions of personal control over school outcomes [2], and enhance self-regulatory skills and knowledge [2, 4]. Students whose parents are involved with their school earn higher test scores [1, 5, 6], receive better grades [7 – 9], show improved social skills [5, 8, 10], have increased school attendance [7], have lower high school dropout rates [11], and have lower reported behavioral problems [3, 8].

Although few studies have examined parent–school involvement for parents of children in special education, there is evidence of a positive impact of such involvement on parents and students. For example, one study showed that mothers of children with developmental disabilities had significantly lower levels of stress when they had a good relationship with their child’s school [12].

Researchers’ recommendations to promote open communication between parents and schools emphasize malleable factors such as building rapport, developing plans tailored to each family, and actively including parents [13 – 16]. Despite such recommendations, parents of children with special education needs often face barriers that prevent them from active school involvement. One barrier often identified by parents of children in special education is feeling stigmatized by school personnel. For example, such parents often report feeling blamed for their children’s behaviors and therefore becoming apprehensive about expressing opinions [17]. Further, parents of children in special education report that, when they do advocate for their child, schools often express a deficit view of their child and do not look at their child’s positive qualities [17, 18]. This deficit view perceived by parents often leads to conflict with the schools [18] and many parents report they do not know how to advocate for their child without becoming adversarial [19, 20].

### 1.1. The Current Study

Participants in this study are a subset from the Students With Involved Families and Teachers (SWIFT) intervention development study, a program to support students with EBD transitioning from treatment to less restrictive school settings [21, 22]. Parent–school communication emerged as a critical component of student transitions over the course of the SWIFT study, with communication challenges representing a key malleable barrier to successful transitions [21]. In the current study we examine parent–school involvement broadly by soliciting parents’ and teachers’ explanations of how and when they communicate with each other. We conducted semi-structured, qualitative interviews with parents and teachers of students with EBD to ask about their parent–school communication experiences. The qualitative interviews were guided by these research questions:

1. What does parent–school communication currently look and feel like?
2. What barriers do parents and teachers face related to effective communication?
3. How would parents and teachers define their ideal method, topic, and frequency of parent–school communication?

## 2. METHODS

### 2.1. Participants and Setting

We used maximum variation purposive sampling [23] to capture a range of perspectives from parent and teacher informants involved with students receiving special education services for EBD at a day-treatment school (DTS) in the Pacific Northwest of the US. As noted above, parent and teacher participants were a subset of the SWIFT study [21, 22]. All participants were recruited by a study representative and participated in an in-person Institutional Review Board approved informed consent procedure. Participants gave written consent to participate in the research study.

#### 2.1.1. Parents

Eight parents of students attending the DTS (*n*=3) or who had attended the DTS in the past 6 months (*n*=5) participated in the qualitative interviews. Three were from the SWIFT intervention condition, and five were from the control condition. All parents identified as white. Seven participants were biological mothers of the target child, and one was an adoptive father. Parents’ education included high school (*n*=3), community college (*n*=2), 4-year college (*n*=2), and graduate school (*n*=1). Household income ranged from $20,000 to over $100,000.

#### 2.1.2. Teachers

Seven teachers (*n*=4 female; *n*=3 male) participated. Six teachers identified as white, and one identified as multiracial. They taught in both general education classrooms (*n*=3) and special education classrooms (*n*=4), but all had a student in their classroom who attended or had attended the DTS within the past 6 months. The teachers taught in public middle or high schools (*n*=4), a military charter school (*n*=1), a private treatment school for students with Autism Spectrum Disorder (*n*=1), and the DTS (*n*=1). Years of teaching experience ranged from 1–31 years.

#### 2.1.3. Interviews

We conducted 15 individual semi-structured in-depth qualitative interviews. Questions were designed to solicit descriptive information about the parent–school communication experiences of the participating parents and teachers. We asked parents: (a) to describe their current communication with schools, including methods (e.g., email, phone, inperson), topics, time spent communicating, and who initiates conversations; and (b) to identify barriers to parent–school communication. Teachers were asked parallel questions about their experiences with parent–school involvement.

### 2.2. Analysis

All interviews were audio-recorded and transcribed verbatim. We verified all transcripts by comparing the audio to the text and entered bracketed notes to provide context to the transcripts. The second author conducted the coding of all transcribed interviews by assigning portions of text to one or more codes. She developed a codebook throughout the coding process with new codes added or modified over time and compared all transcripts against the final codebook. Both authors participated in a peer debriefing process to choose representative quotes for the results section.

## 3. RESULTS

Themes regarding current communication experiences, as well as barriers to communication, emerged from the interviews. Parent–school communication varied by the type of school students were attending (e.g, day-treatment school, neighborhood school, military charter school, or private treatment school), while barriers identified were more consistent across settings. Suggestions to improve communication also emerged throughout the interviews.

### 3.1. Current Communication

Sub-themes for each setting—day-treatment school, neighborhood schools, a military charter school, and a private treatment school—emerged as parents and teachers described current communication.

#### 3.1.1. Day-Treatment School

All parents of children currently attending the DTS reported contentment with their school communication, and four of the five parents who no longer had a child at the DTS reported positive memories about communication with the DTS staff. Three consistent sub-themes emerged as parents discussed positive communication experiences with staff at the DTS, including: (a) Parents felt that teachers cared about talking with them, (b) parents felt involved in the planning, (c) and parents felt the use of a daily point card was helpful.

First, parents in the sample reported they felt the staff at the DTS made an effort to talk with them. They felt teachers cared that parents knew what was happening at school and cared about home circumstances. For example, one current DTS mother mentioned that her son’s teacher called at the beginning of the school year just to check in: “It seemed like they were really interested in how his summer went and how...things have been going and just a check in...It was nice to have him really show an interest.” A former DTS mother echoed similar feelings about the way that the DTS teacher reached out to her: “…we definitely made sure we had some communication...I write things every day and they’d send writing back.” Several parents mentioned that they felt the DTS staff were always open to communication. One parent summed it up by saying, “I think the staff is always available for me to ask questions, or they’ve always been very reasonable to get a hold of and very prompt.”

Second, all but one of the parents in the sample shared stories that showed they felt involved in planning at the DTS. One mother described a time when her son had a negative experience at home and then acted out in class the next day. The mother reported that the teacher contacted her to talk about the incident and set up a plan to regularly communicate: “We decided that anytime something would happen like that, that we’ll communicate it so that she knows that he’s been through something that could possibly trigger his, you know, anxiety, or anger…”

Third, parents felt that the point card at the DTS was a helpful communication tool. The DTS point card includes daily teacher ratings of student behavior with specific comments for the student and his/her parents. DTS students bring the point card home each day and return it to the teacher with parent signatures and written comments. Many parents, both past and current DTS parents, felt that the DTS point card was an effective tool to find out about their child’s school day and promote regular communication with their child about school. For example, one current parent reported that, “What my child tells me how his day went, it’s not necessarily what the point card reiterates. But then again I can ask what happened in this class and why did you, you know, get a zero for this?” Several other parents echoed this same idea. Another current DTS parent explained that the point card helped him talk to his daughter about her behavior at school, “...because the few times she has been marked down I can talk to her about that.”

#### 3.1.2. Neighborhood Schools

When parents discussed the transition to the neighborhood school and new staff, they reported a range of experiences and feelings. One mother reported satisfaction that the neighborhood school continued to use the daily point card from the DTS. Although the neighborhood school did not regularly implement daily point cards, staff included a point card as a component of the behavior support plan in the student’s individualized education plan (IEP). As a result, the mother said she felt the school personnel were listening to her and understood her family’s needs. She discussed feeling included in the decision making at the new school and felt that the school personnel respected her family as they listened to her concerns and implemented solutions.

Another mother reported that, upon transition to the new school, she received very little information about her son: “I know it gets hard because they have so many students, but I know not all of them need as much attention as my son. So it would be nice if I could get an email or something.” This mother seemed fatigued throughout the interview, saying she wanted more information from the school about her son, but felt that she would need to go out of her way to get it. She wished the school had something in place already to help her.

The third mother who transferred to a neighborhood school was the only parent in the sample who identified that she was not content with the communication at any of her child’s prior schools. She reported negative feelings about the school system in general, saying she felt the schools did not respect her input. Although she discussed communication challenges at the current and prior schools, she did identify that one teacher at her son’s new neighborhood school communicated effectively with her, saying, “They’re very responsive to me and take my individual considerations into consideration, like what I think. [The teacher will] call and say, ‘This isn’t going well. What do you think we could do?’” This mother’s comments and affect throughout the interview suggested that she had a history of frustration with the school system because she felt judged and that her input was not valued. Though things were better at the time of the interview than they had been, she still wished for more respect and inclusion from school personnel, saying, “I have reached out to the Autism Consultant and she never got back to me. I’ve tried to include the ICC [Interagency Coordinating Council] people, and they never got back to me. I wish it was more like a team effort.”

#### 3.1.3. Military Charter School

Following the transition from the DTS, one mother sent her child to a military charter school after significant frustration about communication with the neighborhood school:

I don’t think that communication was good at all. They waited. [My son] had a point card, and he would bring the point card home, and it would be signed that he was doing well...And then a month went by. That’s when they called me and said he wants to be in the office all the time...when he should’ve been in class learning...But they waited for like a month.

The poor communication coupled with the difficulty her son was having at the neighborhood school led this mother to enroll her child at a military charter school. She reported that she felt the same sort of positive relationship with the charter school that she’d had at the DTS. When talking about a meeting, she said,

I think that they were all thinking that we are on the same page. And they’re there for the kids, I felt like. I felt kind of safe like I did with the [DTS] because I feel like they have the tools to help them with their behavior and to help them when they have a certain. If they’re angry, to help them deal with certain things. I feel safe when he’s there.

This mother said she felt her opinion was heard at both the DTS and the military charter school, that she was involved in the decision-making, and that the school cared about her family.

#### 3.1.4. Private Treatment School

One mother elected to send her child to a private treatment school after he completed the DTS program. She felt satisfied with the various programs the new school had, saying, “So far we’ve had, you know, nothing but good vibes from them.” However, this mother still wondered why the communication was not more like it was at the DTS, saying,

…we’ve gone there [private school] for a couple open houses, and we’ve been there for the IEP, and we’ve done a little communication over the computer where they sent home some homework through his computer. Um, however, other than that we really haven’t had a whole lot of conversation and communication. That’s something that’s important to us, to have communication with the schools. The last school that he went to we definitely made sure we had some communication.

She later mentioned that at her next scheduled IEP meeting she was going to ask why they were not communicating more regularly. Though this parent felt comfortable with the school and comfortable advocating for more communication, she was confused about why communication was not automatic.

#### 3.1.5. Teacher’s Views on Communication with Parents

Most teachers reported that they had a system in place to give parents school-related information about their child. However, teachers used different types of systems to accomplish this goal. For example, one neighborhood school teacher reported that he used a code system in his online grade-book to indicate whether the assignment was submitted on time, never turned in, or turned in and received a failing grade. Another neighborhood school teacher said that she sent out weekly emails to all parents with information about homework expectations and scheduling updates. Most teachers reported that they updated their online grade-books regularly and hoped that parents checked them. Many teachers also set up 1:1 parent meetings as well as open houses and conferences. Unfortunately, most of the teachers felt that their systems for communication were not fully utilized by parents. One teacher discussed his confusion with parents’ lack of communication, saying,

I have a son... as soon as I saw a grade below like a “C” I would be calling the teacher. A lot of parents don’t do that because either they don’t know or, like, I don’t know. I’m like, they send home progress reports. Don’t [parents] look at the progress reports and have questions?

Another teacher said that he often felt dismissed by parents when he tried to reach out, saying, “It’s usually a very quick conversation, and they’re quick to...get me off the phone...I feel like I’m being blown off. It’s like they don’t have time to take the phone call.”

Despite parents’ general preference for communicating with the DTS compared to other schools, the DTS teacher we interviewed did not report significantly different communication habits from other teachers. However, similar to the parents, the DTS teacher mentioned finding the daily point card useful for communicating with parents, compared to the online grade-book favored by teachers in other settings.

### 3.2. Barriers to Communication

In addition to the challenges noted above, six specific sub-themes emerged as parents and teachers identified barriers to effective parent–school communication. These themes include technology, institutional issues, time, parents’ lack of interest, and school proximity.

#### 3.2.1. Technology

Multiple interviewees mentioned technology as a barrier to parent–school communication, but most participants listed it as a hypothetical barrier and not one that they had experienced. Teachers said some parents cannot access a computer to check emails and the online grade-book. One mother said technology may explain why the Autism Consultant never returned her email.

#### 3.2.2. Institutional Issues

Two parents said that they noticed institutional-level barriers—some structural and some cultural—that prevent school personnel from being able to effectively communicate with parents. One mother, who had a child at the DTS at the time of the interview, reflected back on the issues she had at two schools prior to the DTS:

...I think the schools prior to [DTS] didn’t have the ability to work with me. [The schools] really tried…because I don’t know if I was a teacher I’d be able to handle behavior issues on top of the kids that you’re trying to teach.

Another parent echoed the idea that schools might not have the infrastructure or culture to effectively work with parents saying, “They’re pretty set in their ways of how they do things. My whole idea of like a team approach—that doesn’t really happen. So that’s just too much to even go there.”

Teachers also identified institutional-level barriers that they face when trying to effectively communicate with parents. One teacher discussed how schools communicate with parents about problems but not positives, saying, “We, as the... institution. I don’t know if communication with home is always super positive. Like it’s...’Oh, if there’s something that happens that’s not positive, let’s call home then.’” Another teacher described the difficulty working with parents who have had negative experiences communicating with schools:

So if you’ve [the parent] had not very fabulous conversations with teachers in the past, you bring that energy into the new conversation...That protection or the defensiveness, I find that to be a difficult thing. You have to kind of build that trust back up.

A third teacher described her fear of proactively talking to parents about their children’s behavior problems because their communication with schools may have already been dominated by negative information about the student. She explained that she did not want to worry parents and therefore intentionally avoided communication about small problems, saying,

…Just because they’re struggling now, I don’t want [the parents] to freak out that, “oh my gawd, they’re not going to graduate high school” or something. Because some parents jump to like the worst conclusion, and I don’t want to, um, force that on them.

Two teachers reported they wished the school utilized existing parent–school communication resources more effectively. One said, “Like there’s an open house at the beginning of the year. I don’t think it’s designed as effectively as it could be.” She went on to say that she wished the open house were an opportunity to better communicate expectations with parents for the upcoming school year, but has not seen that happen. Another teacher mentioned that many teachers do not update their online grade-books regularly, so checking grades is frustrating for parents.

Several teachers mentioned cultural barriers that contribute to parents feeling less comfortable in the school given the institutional structure or school culture. One teacher said, “Unfortunately in this district, we have a lot of poverty, and we have a lot of students that just flat out say...’My parents don’t need this [education] so I don’t need this’…” Two teachers discussed ways they try to remedy cultural barriers but noted limited success due to long-held school traditions or school culture. One teacher said that, although he typically goes out of his way to help parents feel comfortable at IEP meetings, other school personnel make that difficult:

I try to always make the tone loose and comfortable, but sometimes it doesn’t work out that way. And it depends on the school too, because there are some district reps that’ll just kinda go with [the loose and comfortable tone] and then those other district reps that really wanna do it this [formal] way.

Another teacher said that he has advocated having more at-ease parent teacher conferences:

One of the things I brought up in the past with the administrator was kind of have a casual night for us, like we just wore sweatshirts and jeans, and everybody else could come the same way, and I thought that’d be very comforting for all people to come in and feel like they don’t have to dress up to come to these parent-teacher conferences...Sad thing was nothing was ever done.

When asked why he thought the administrators did not implement casual parent-teacher conferences, he said, “There is a pattern I think for everything. And I think they just get used to the pattern and...change is hard for a lot of teachers.”

#### 3.2.3. Time

Five teachers and five parents mentioned time constraints as a barrier. All five of the parents said that they assumed the teachers are too busy for more communication. Three parents also mentioned that they are personally busy.

All five of the teachers who identified time as a barrier to parent–school communication said that they themselves are too busy for more communication, and three teachers mentioned that parents have busy schedules, as well. The DTS teacher said that although the point card was a part of his daily schedule, communication could be even better if teachers had extra time to leave detailed notes on the card:

It’s challenging sometimes though when you’re trying to leave a detailed note while six students are over here, starting to spin, you know? …I wish…there was more time to leave more detailed and more meaningful notes instead of… just the “Great job!” signed, who’s next?

Other teachers mentioned time constraints due to large class sizes, being over-worked, and not having adequate time to communicate with parents. For example, while discussing her preference to have more communication with parents, one teacher explained that she relied on the parents checking the online grade-book and her weekly emails but had limited time for more parent communication, saying, “… I have a hundred and sixty kids in the day, and I don’t know how I would manage [more parent communication] … ideally yeah, we would be able to do that…but there just isn’t time in the day.”

#### 3.2.4. Parents’ Lack of Interest

Five teachers identified parents’ lack of interest as a barrier to parent–school communication. Teachers discussed their frustration with parents who seem uninterested in communicating despite a variety of available mechanisms and how they wanted the parents to take advantage of these systems. One teacher said,

I think a lot of parents send their kids off to school and expect them to do their thing and everybody to kind of fix it here [at the school]. So, I think requiring that the parents be involved and at least acknowledging and reading [newsletters or gradebooks].

Another teacher wished that parents were more involved in the systems already in place and tried “to attend more parent-teacher conference meetings, to attend more open houses, to be more part of the community, and try to get involved more.”

Two parents mentioned their own lack of interest in more regular communication with the school. One parent summed it up by saying, “I think that the biggest barrier probably is me getting in to see the teacher. If I need communication, I should be requesting.”

#### 3.2.5. Teachers’ Lack of Interest

Two parents mentioned that teachers seemed uninterested in getting too involved with parents. One parent said, “Maybe they don’t want to deal with parents.”

Two teachers also mentioned that in certain situations, they do not want to interact more with the parents. One teacher said, “I do have a few helicopter moms, and that can be equally as frustrating as a parent not communicating.” This teacher told about story of a mother who was continually calling to ask about her middle schooler’s grades and assignments without having her child take responsibility for completing the work. Another teacher reported that she felt that parents, at times, were disrespectful of teachers’ time. She identified that she wished that parents would speak more concisely: “I think being specific about—and respectful—of time. Often when I find myself getting frustrated…it’s because there’s a long story that doesn’t have anything to do with the moment...Because then it becomes a counseling session for the family.” In neither of these cases were the teachers opposed to communicating with families; they just felt that some parents communicated excessively.

#### 3.2.6. School Proximity

Two parents mentioned the lack of proximity to the school as a barrier to effective parent–school communication. Both parents lived a considerable distance from the day-treatment school and had their children bussed to school. These parents felt that that although they had been able to stop by their neighborhood schools and chat with teachers before, the distance to the new school was a barrier to regular, informal meetings with teachers.

## 4. DISCUSSION

The parents in this study had children with EBD who were currently or had recently received special education services from a day-treatment school, and the teachers were special education and general education teachers working with the same population. The experiences and feedback from the interviews were informed by participants’ experiences supporting students with EBD in schools.

Parents reported greater overall satisfaction with the method and tone of communication at schools where they felt informed about their child’s progress and included in planning and decision making. Parents emphasized key factors—including that teachers cared to talk to them, involved them in planning and valued their opinions, and communicated regularly—as facilitators to effective parent–school communication. When children transitioned to new schools, that feeling of involvement in planning and decision making, as well as comfort communicating with teachers, was often diminished as parents navigated new relationships and communication methods with new teachers and school staff. At the neighborhood, military charter, and private treatment schools, parents were learning each new teacher’s communication preference and feeling welcomed and included in these new settings to varying extents. As for the teachers, most also highlighted the importance of and desire to have effective communication with parents. Teachers actively engaged in various methods of communication in an effort to reach out to parents and described specific strategies, including daily point cards, weekly emails to parents, and up-to-date online gradebooks. However, many teachers also reported feeling confused or frustrated when parents were not engaged with or utilizing these communication strategies.

Participants also discussed a range of barriers to effective parent–school communication. Barriers included both practical limitations, such as a lack of time, as well as broader, more structural or cultural limitations. Both parents and teachers raised complex institutional or school culture dynamics that contribute to tensions with parent–school communication. Specifically, they felt that because schools often communicate primarily about problems, it would require significant effort to welcome all parents and win their trust—especially parents with negative histories with schools, who might either bring that frustration into the new school setting or shy away from communication opportunities. Three broad recommendations to address malleable parent–school communication barriers for parents and teachers of students with EBD emerged from the study findings.

First, findings from the interviews suggest that teachers provide parents with detailed information about consistent parent–school communication methods. Regular, predictable communication with school has been shown to decrease parent stress for parents of students with disabilities [12]. Parents in the study described the many benefits of knowing how to effectively communicate with teachers and highlighted the challenges that arose when they were unsure about how best to approach teachers. In addition, providing parents and teachers with a consistent, effective communication strategy could serve to mitigate barriers related to lack of time.

Second, both parents and teachers identified proactive, regular parent–school communication about student problems at home and school as an effective way to intervene early when problems arise. In addition, both parents and teachers identified the need for schools to more regularly communicate with parents about their child’s strengths and positive behaviors at school. Parents in the study reported that when teachers reached out to them, they felt like the teachers cared about their family. Proactive communication, balancing reports of both student problems and positive feedback, could not only serve as a strategy to build parent-teacher rapport [15], but also promote active parent involvement in developing school-based behavioral support plans [15, 16].

Third, consistent with prior research [13 – 16], parents and teachers suggested ways that schools can welcome more active parent involvement. In addition to the proactive parent–school communication described above, many of the teachers highlighted the need for changes to the school culture, including ways of interacting with parents and involving them in decisions. Specifically, teachers identified the need for school staff to be welcoming and approachable to parents from a range of socio-economic and educational backgrounds. Parents and teachers suggested strategies to build this rapport, including casual dress for parent meetings, emphasizing a team approach that includes the parent’s input, and an increased focus on what students are doing well.

## 5. LIMITATIONS AND FUTURE DIRECTIONS

Parent participants were drawn from one DTS in the Northwest of the United States. The participants were predominantly white. However, there was a range in the income and educational attainment of the parents. Conducting further interviews with parents and teachers from diverse ethnic and racial backgrounds and in other geographic regions could increase generalizability of findings. Additionally, the interviews for the current study focused on parent–school communication, which is only one element of parent–school involvement. Qualitative interviews with parents and teachers about current experiences with and barriers to other forms of parent–school involvement (e.g., volunteering in the classroom or participation in after-school activities) would broaden our understanding of parent–school involvement as a whole.

## CONCLUSION

Consistent with research on parent involvement for families of students receiving special education [12], the parents and teachers in our study identified parent–school communication as a critical factor for the educational success of students with EBD. The three broad recommendations to increase effective parent–school involvement that emerged from the results focus on malleable factors. Although static barriers like a parent’s proximity to the school cannot be addressed via intervention efforts, strategies to increase parents’ knowledge about effective parent–school communication methods, proactive communication about both problems and progress, and attention to a welcoming school culture are potential intervention targets.
